# Supplementary material for: Comprehensive profiling of social mixing patterns in resource poor countries: A mixed methods research protocol
Source: PLoS One. 2024 Jun 24;19(6):e0301638. doi: 10.1371/journal.pone.0301638 (PMC11195963; doi:10.1371/journal.pone.0301638)
Supplement: S1 File — Survey: Enrollment survey Electronic contact diary Exit survey Paper contact diary Places diary Focus group discussion guide Cognitive interview guide. (DOCX) [file pone.0301638.s001.docx]

Inclusivity in global research

PLOS’ policy on inclusivity in global research aims to improve transparency in the reporting of research performed outside of researchers’ own country or community and ensures that PLOS publications reporting global research adhere to high standards for research ethics and authorship. Authors of relevant research articles may be asked to complete the questionnaire below, which outlines ethical, cultural, and scientific considerations specific to inclusivity in global research. This questionnaire may be requested when researchers have travelled to a different country to conduct research, if research uses samples collected in another country, research with Indigenous populations or their lands, or if research is on cultural artefacts. Researchers travelling to another country solely to use laboratory equipment will not normally be required to complete the questionnaire. However, the questionnaire can be requested at the journal’s discretion for any submission – if you have been requested to complete this questionnaire by the PLOS journal you submitted to, please do so.

Please complete the questionnaire below and include this as a Supporting Information file with your manuscript. Note that if your paper is accepted for publication, this checklist will be published with your article in the supporting information files. Please ensure that you reference the checklist in the main body of your manuscript. We suggest adding a subsection ‘Inclusivity in global research’ to your Methods section and adding the following sentence: “Additional information regarding the ethical, cultural, and scientific considerations specific to inclusivity in global research is included in the Supporting Information (SX Checklist)”

The questions have been designed to be applicable to a wide range of study types, and there are subsections for both human subjects research and non-human subjects research. If any of the questions are not relevant to your research please mark them as “N/A” as appropriate.

**Ethical considerations, permits and authorship**

*This section is applicable to all research types.*

Provide details as to who granted permissions and/or consent for the study to take place in the Methods section of your manuscript. This should include the names of **all** ethics boards, governmental organizations, community leaders or other bodies that provided approval for the study. If individuals provided approval refer to these people by their role or title but do not list their name(s).

Reported on page number: pages 18 & 19

If there were any deviations from the study protocol after approval was obtained please provide details of these changes in the Methods section of your manuscript.
Did this study involve local collaborators that are residents of the country where the research was conducted or members of the community studied? If you do not have any authors from said communities, please provide an explanation for this below.

Reported on page number: n/a

Yes, local collaborators that are residents of the country where the research was conducted or members of the community studied were involved in all stages of the study.

Everyone listed as an author should meet PLOS’ criteria for authorship and all individuals who meet these criteria should be included in the author byline, rather than the acknowledgements. For further information please see the journal’s Authorship Policy.

**Human subjects research (e.g. health research, medical research, cross-cultural psychology)**

Did you obtain written informed consent from a representative of the local community or region before the research took place? How did you establish who speaks for the community? Details of written informed consent obtained from study participants should be reported separately in the Methods section of your manuscript.

The study team includes co-investigators and field team members who are indigenes and researchers from institutions at the various sites.

Appropriate strategies were used to recruit participants to each site. First, we held meetings with local relevant stakeholders such as local authorities (chiefs of administrative posts and localities, heads of the villages, chiefs of neighborhoods or any other authority), religious leaders, and health practitioners to explain the purpose of conducting this study and identify potential participants. The dates and venues of meetings were defined according to the availability of the participants and according to the epidemiological situation and COVID-19 alert system of the Ministry of Health for the various sites.

How did members of the local community provide input on the aims of the research investigation, its methodology, and its anticipated outcome(s)?

We conducted formative phase (focus group discussions, cognitive interviews, and pilot studies) to understand contextual factors and attributes of the community associated with the feasibility and acceptability of paper diaries and wearable proximity sensors in each site. This understanding informed the design of data collection tools and implementation of the survey and other relevant study procedures.

When engaging with the local community, how did you ensure that the informed consent documents and other materials could be understood by local stakeholders?

We conducted formative phase (focus group discussions, cognitive interviews, and pilot studies) to understand contextual factors and attributes of the community associated with the feasibility and acceptability of paper diaries and wearable proximity sensors in each site. This understanding informed the design of data collection tools and implementation of the survey and other relevant study procedures.

During cognitive interviews, we clarified through a few carefully selected questions on how community members understand and interpret the consent form, survey questions and procedures, specifically what these questions mean in the local context. The interviews helped to ensure that consent forms and other study tools achieved their measurement purpose and elicited the intended meaning of the question. With the assistance of the team anthropologist, we developed a cognitive interview guide that included select survey and contact diary questions that were difficult to understand during FGDs. The cognitive interviews identified comprehension and language problems, i.e., not knowing the meaning of words/phrases, or having different local or cultural terms that are more appropriate or better understood; inclusion/exclusion problems, i.e., determining whether certain concepts are to be considered within the scope of an item, and whether caregivers include or exclude experiences within the intended scope of the question; temporal problems, i.e., the period to which the question applies; logical problems, e.g., how participants interpret certain phrases in survey questions, and computational problems, e.g., readability of items, and guessing age of contacts. The interviewer starts by obtaining written consent from participants. Participants were then asked to read each question, and before responding with an answer, they paraphrased the question in their own words for the interviewer to ensure that they understood the meaning correctly. While answering the question, they could also ‘think aloud’ to share what was going through their mind when they are considering the response options. Participants were then asked to complete a template of actionable recommendations based on the interviews.

Will the findings of the research be made available in an understandable format to stakeholders in the community where the study was conducted (e.g. via a presentation, summary report, copies of publications, etc.)? Please provide details of how this will be achieved.

The results of the study will be shared with Ministry of Health and local authorities (district administration as well as with the district directorate of health).

**Non-human subjects research using specimens/ animals collected as part of the study, or those housed in archival collections. Examples include archaeology, paleontology, botany and zoology.**

Did the permission you obtained from a local authority to perform the study include an agreement on access to outputs and benefit sharing? This may include procedures to enable fair distribution of the benefits and resources arising from the research performed. Please include any details of Prior Informed Consent and Benefit Sharing Agreements obtained. These may be required by field-specific regulations, for example the Convention on Biological Diversity (CBD) and the associated Nagoya Protocol.

n/a

If the material used in your study was imported, please A) provide the year it was imported and B) indicate whether permits were obtained to import/export the materials used, C) provide details of any permits obtained. If this information is not available, please indicate this.

n/a

If you used archival specimens, please state how the material used in your study was acquired by the institute it is held in and provide details of any permits obtained for the original excavations/ sample collection. If this information is not available, please indicate this.

n/a

How was the potential cultural significance of the materials collected in your study to local communities considered in your research design? Were Indigenous peoples and/or local researchers and institutions involved with archaeological excavations / collection of specimens? If so, please provide a description of their involvement.

n/a

If your manuscript includes photographs of human remains please indicate whether authors obtained permission from descendants or affiliated cultural communities to do so.

n/a
